# Collective Educator Efficacy in Medical Education: A Conceptual Synthesis and Framework for Program Evaluation

**DOI:** 10.12688/mep.21825.1

**Published:** 2026-09-12

**Authors:** Pritam Biswas, T K Sukumar, Atuhaire Arnold

**Affiliations:** 1 Medicine, St Matthew's University School of Medicine, George Town, Grand Cayman, Ky11204, Cayman Islands; 2 Microbiology and Immunology, St Matthew's University School of Medicine, George Town, Grand Cayman, Ky11204, Cayman Islands; 3 Internal Medicine, Kabale University School of Medicine, Kabale, Western Region, 40601, Uganda

**Keywords:** collective educator efficacy, complex adaptive systems, faculty development, medical education, program evaluation

## Abstract

**Introduction:**

Program evaluation in medical education often emphasizes outcomes such as examination performance, competency attainment and learner satisfaction, but these indicators do not fully explain why programs with similar structures can produce different educational results. This review introduces collective educator efficacy as a potentially important but underexamined determinant of program implementation and program quality.

**Methods:**

We conducted a structured narrative review with conceptual synthesis, drawing literature from medical education, collective efficacy research, organizational psychology, faculty development and program evaluation. The review used transparent search and selection procedures and was guided by published standards for high-quality narrative reviews.

**Results:**

The literature suggests that educational outcomes are shaped not only by curriculum design, but also by faculty coordination, shared expectations, psychological safety, assessment calibration and the collective capacity to implement, adapt and sustain educational practices. Foundational work on collective efficacy shows that shared group efficacy beliefs are associated with performance and persistence, while adjacent health professions education literature on programmatic assessment, faculty development, interprofessional learning and communities of practice highlight the importance of shared mental models and collaborative culture. Synthesizing these literatures, we define collective educator efficacy as a faculty group’s shared belief in its capability to work together to achieve educational goals and propose a framework for evaluating it within medical programs.

**Discussion:**

A collective educator efficacy-informed approach may help explain variation between structurally similar programs and provide a practical lens for program improvement, faculty development and evaluation design. Empirical work is now needed to operationalize and validate the construct in medical education.

## Introduction

Program evaluation in medical education traditionally focuses on learner outcomes, curricular structures, and individual teaching performance. Standard evaluation frameworks emphasize examination results, competency attainment, accreditation benchmarks, and learner satisfaction. While these dimensions are essential, they often fail to explain why programs with comparable curricula, resources, and faculty credentials demonstrate markedly different levels of effectiveness.

Program evaluation in health professions education must do more than determine whether a program “worked.” Contemporary evaluation scholarship argues that educational interventions are enacted within complex social systems, and that useful evaluation therefore requires attention to context, mechanisms, implementation and local adaptation, not outcomes alone. This matters in medical education because programs that appear structurally similar on paper may function very differently in practice depending on how faculty interpret standards, coordinate teaching, enact feedback, and respond to emerging challenges. ^1^ ^–^ ^5^

Medical education operates within complex social systems in which faculty members function not as isolated instructors but as interdependent contributors to shared educational goals. Systems perspectives in health professions education emphasize that outcomes emerge from interactions among individuals, structures, and environments rather than from discrete curricular components alone. ^1^ ^,^ ^2^ Despite this recognition, program evaluation rarely examines the collective psychological and cultural conditions of the faculty who enact curricula, design assessments, and support learners. As a result, a critical explanatory layer remains underdeveloped. While constructs such as faculty engagement, organizational culture, and learning climate have been explored in medical education, these frameworks primarily address values, behaviors, or motivation rather than shared beliefs about collective capability. The concept of collective efficacy, which has been extensively studied in school education, has not been explicitly translated to faculty groups in medical education contexts. To our knowledge, medical education literature has not yet explicitly articulated a faculty group-level construct focused specifically on shared beliefs about collective educational capability within program evaluation. As a result, the role of shared faculty beliefs about their joint capacity to influence learner and program outcomes remains underexamined in current program evaluation models.

Two medical schools may implement similar competency frameworks, assessment systems, and curricular maps, yet one demonstrates cohesive implementation, consistent assessment standards, and strong learner progression, while the other struggles with fragmentation, uneven faculty engagement, and inconsistent learner outcomes. Structural explanations alone are insufficient to account for such divergence. The difference may lie not only in what faculty know or how programs are designed, but in what faculty believe they can accomplish together.

One plausible explanation for this variation is that educational structures do not implement themselves: faculty groups do. In programmatic assessment and competency-based training, educational quality depends on shared understanding of standards, credible collective judgement, psychologically safe feedback cultures, and consistent faculty engagement across teaching and assessment activities. Evidence from health professions education shows that shared mental models improve group decision making, that competency committee decisions are influenced by group dynamics and bias risks, and that implementation of programmatic assessment depends on collective understanding and faculty support. These findings suggest that faculty group functioning is not peripheral to program evaluation; it is part of the mechanism through which programs succeed or fail. ^6^ ^–^ ^9^

This review proposes Collective Educator Efficacy (CEE) as a systems-level construct that addresses this gap. CEE is defined as the shared belief among a defined group of faculty that, through coordinated effort, can positively influence learner outcomes and the overall effectiveness of their educational program. Adapted from Bandura’s theory of collective efficacy ^10^ ^–^ ^12^ and research on collective teacher efficacy in general education, ^13^ ^,^ ^14^ CEE offers a theoretical lens for understanding how faculty belief systems shape program functioning beyond curriculum design alone. The theoretical basis for this construct comes from the literature on collective efficacy, which describes a group’s shared belief in its conjoint capability to organize and execute action toward desired goals. In educational settings, collective efficacy has been associated with performance, persistence and positive work-related outcomes, and higher education research suggests that collective efficacy can be meaningfully examined among college instructors as well as schoolteachers. Taken together, this literature supports adapting the construct to medical education, where teaching, assessment and curriculum implementation depend on coordinated faculty work rather than isolated individual action. ^10^ ^,^ ^11^ ^,^ ^13^ ^,^ ^15^ ^–^ ^17^

By situating faculty for collective beliefs within the ecology of program evaluation, CEE provides a conceptually distinct way to explain variation in program effectiveness across similar structural contexts and the proposed conceptual model is shown in Figure 1.

**Figure 1.**
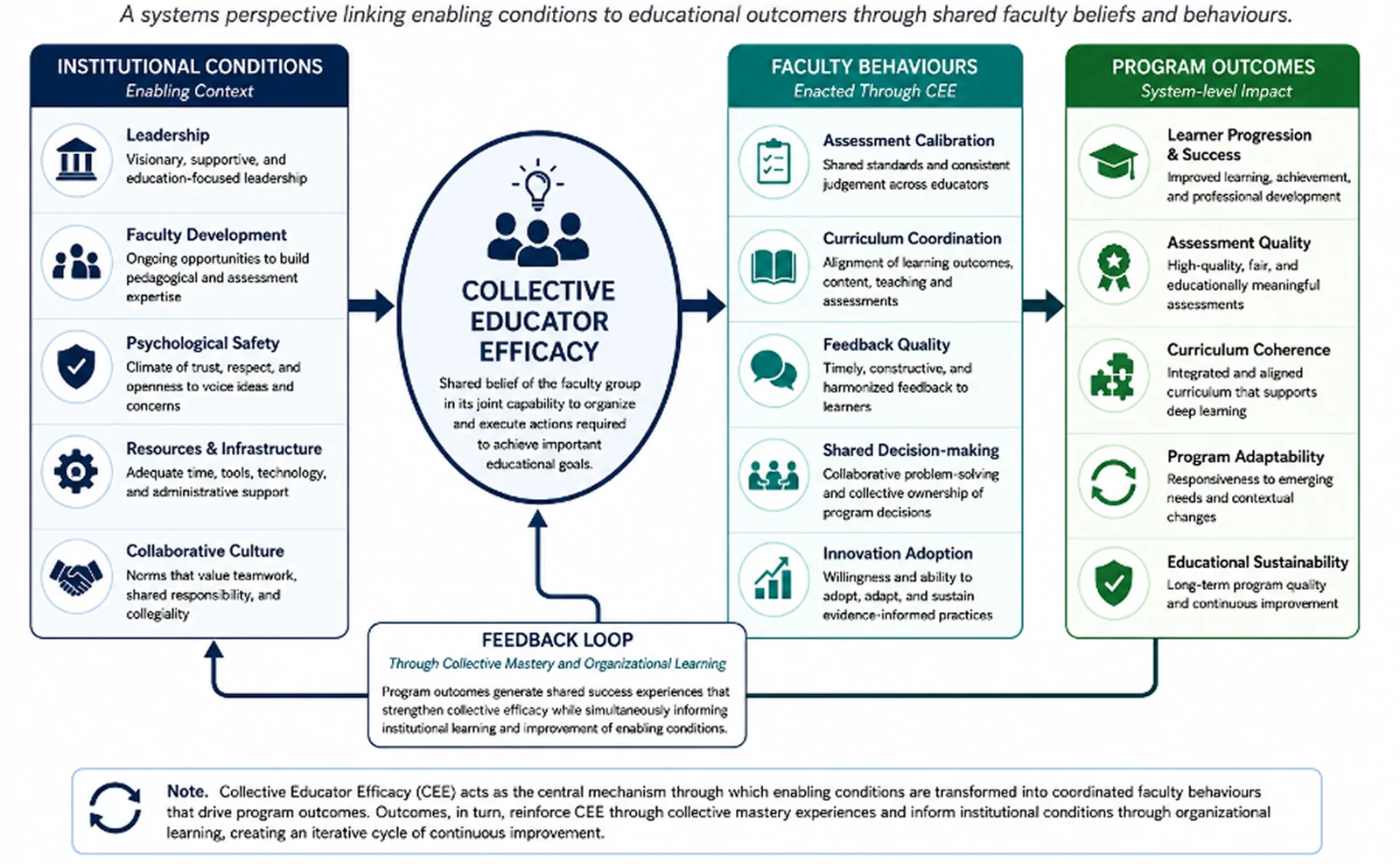
Conceptual model of Collective Educator Efficacy (CEE) in medical education.

This author-developed conceptual framework illustrates CEE as the central faculty group–level mechanism linking enabling institutional conditions to educational outcomes. The model depicts a directional pathway in which contextual enablers give rise to shared collective efficacy beliefs that translate into coordinated faculty actions, ultimately contributing to program-level outcomes. The dual feedback loop reflects an iterative process whereby educational outcomes reinforce CEE through collective mastery experiences while simultaneously informing institutional conditions through organizational learning and continuous quality improvement. The framework synthesizes concepts from social cognitive theory and contemporary medical education scholarship (Bandura, 1997; Goddard et al., 2000; Tschannen-Moran & Barr, 2004).

## Methods

This manuscript was developed as a structured narrative review with conceptual synthesis. A narrative review design was selected because the literature relevant to collective educator efficacy spans medical education, health professions education, teacher efficacy, organizational psychology, faculty development, and programme evaluation, and therefore includes heterogeneous conceptual, empirical, and methodological sources not readily amenable to a conventional single-effect systematic review. ^18^ ^–^ ^20^

To strengthen methodological transparency, the review was reported with attention to SANRA domains and published guidance for narrative reviews in medical education. ^18^ ^–^ ^20^

Searches were conducted in PubMed/MEDLINE, ERIC, Scopus, Web of Science, PsycINFO, and Google Scholar for literature published from January 1990 to May 2026, supplemented by backward and forward citation tracking of seminal papers.

Search terms combined efficacy-related concepts (“collective efficacy,” “collective teacher efficacy,” “collective educator efficacy,” “faculty efficacy,” “group efficacy”) with medical education and evaluation terms (“medical education,” “health professions education,” “faculty development,” “program evaluation,” “programmatic assessment,” “educational climate,” “implementation,” and “psychological safety”)

We included English-language conceptual papers, empirical studies, reviews, consensus statements, and methodological articles that contributed to understanding collective efficacy, faculty collaboration, educational implementation, or programme evaluation. We excluded sources focused solely on individual self-efficacy without a group-level construct, non-educational clinical efficacy, and learner-only outcomes without implications for educator collectives or programme evaluation. ^18^ ^,^ ^20^

Titles and abstracts were screened by PB and TKS, followed by full-text review for eligibility. Disagreements during title/abstract and full-text screening were resolved by consensus with a third reviewer, AA. Backward and forward citation tracking of key papers was used to identify additional conceptually relevant sources. For each included source, we charted the setting, study design, focal construct, level of analysis, and relevance to the emerging Collective Educator Efficacy framework. We then used iterative thematic synthesis to identify recurrent mechanisms linking faculty collective functioning to programme implementation, assessment, feedback culture, and learner outcomes, and used these themes to develop the proposed framework. ^18^ ^,^ ^20^

## Results

The search and citation tracking process identified a focused body of literature relevant to collective efficacy, medical education program evaluation, faculty development, programmatic assessment, psychological safety, and collaborative learning. After screening for conceptual relevance, 30 sources were included in the final synthesis. Included sources clustered into five overlapping literatures: collective efficacy theory, program evaluation in health professions education, faculty development and organizational change, collaborative decision making and programmatic assessment, and psychological or social conditions that support faculty coordination. The principal literature domains and their contributions to the development of the CEE framework are summarized in Table 1.

**Table 1.** Literature domains informing the conceptual development of Collective Educator Efficacy (CEE) in medical education.

Literature domain	Representative sources	Main Finding/contribution	Relevance to collective educator efficacy (CEE) framework
Collective Efficacy Theory	Bandura, ^10^ ^,^ ^11^ ^,^ ^25^ Goddard et al., ^13^ Tschannen-Moran & Barr, ^14^ Eells, ^15^ Fives & Looney, ^16^ Stajkovic et al., ^17^ Donohoo et al. ^21^	Establishes collective efficacy as a group’s shared belief in its conjoint capability to organize and execute actions required to achieve desired goals. Demonstrates strong associations between collective efficacy, group performance, persistence, coordination, and resilience across educational and organizational settings.	Provides the foundational theoretical basis for conceptualizing educator groups as active determinants of educational implementation, assessment quality, and learner outcomes.
Systems Perspectives in Program Evaluation	Haji et al., ^1^ Frye & Hemmer, ^3^ Gandomkar & Sandars, ^4^ Balmer et al., ^5^ Bleakley, ^12^ Mennin, ^24^ Frenk et al. ^31^	Reframes program evaluation in health professions education from rigid outcome-based metrics toward complex social systems perspectives. Emphasizes the critical role of context, local mechanisms, implementation fidelity, and dynamic adaptation.	Supports the argument that differences in program outcomes may reflect not only curriculum design, but also how faculty groups enact, coordinate, and sustain educational practices.
Faculty Development & Educational Change	Steinert, ^26^ McLean et al., ^27^ Fernandez & Audétat, ^28^ Jenkins et al., ^29^ Steinert et al. ^32^	Positions faculty development as an institutional strategy for organizational culture-building, collaborative practice, and sustained reform, rather than solely an intervention for individual skill acquisition.	Positions CEE as a potential mechanism through which faculty development contributes to shared implementation capacity, educational innovation, and sustained program improvement.
Programmatic Assessment and Group Judgement	Edgar et al., ^6^ Duitsman et al., ^7^ Heeneman et al., ^8^ Schut et al., ^9^ Van Der Vleuten ^22^	Demonstrates that programmatic assessment relies on shared mental models, credible group judgment, multi-rater calibration, feedback literacy, and protected decision-making processes within competency committees.	Illustrates how educator collectives influence assessment coherence, feedback quality, progression decisions, and trustworthiness of program-level judgement.
Psychological safety and Sociocultural Learning	Allen & Boyle, ^2^ Edmondson, ^23^ Senge, ^30^ Lave & Wenger ^33^	Highlights that effective team learning, open communication, feedback exchange, and reflective practice depend on interpersonal trust and psychological safety within working groups.	Identifies social-organizational conditions that may enable or constrain collective educator efficacy within medical education programs.

*Note*: This table summarizes the principal literature domains synthesized in the development of the Collective Educator Efficacy (CEE) framework. It highlights representative literature sources, major conceptual contributions, and their relevance to the construct. Mapping of included literature (n = 30 theoretical and empirical sources; excluding 3 review methodology sources ^18^ ^–^ ^20^) across primary academic domains, detailing core findings and direct theoretical contributions to the CEE framework.

### Theoretical foundations of collective efficacy

Bandura’s social cognitive theory positions efficacy beliefs as central determinants of human agency. ^11^ Self-efficacy refers to an individual’s belief in their capacity to organize and execute actions required to achieve desired outcomes. ^11^ ^,^ ^25^ Individuals with strong self-efficacy set challenging goals, persist in the face of difficulty, and interpret setbacks as opportunities for learning rather than as evidence of failure.

Bandura extended this concept to the collective level, defining collective efficacy as a group’s shared belief in its conjoint capability to organize and execute actions required to achieve goals. ^11^ Collective efficacy is not reducible to the sum of individual confidence. Rather, it emerges from shared mastery experiences, social persuasion, vicarious learning, and collective interpretations of success and failure. ^10^ ^–^ ^12^ Groups with strong collective efficacy demonstrate greater persistence, adaptability, and willingness to engage in coordinated problem-solving.

In general education, collective teacher efficacy has been consistently linked to student achievement. A study by Goddard et al demonstrated that schools with higher collective teacher efficacy achieved stronger academic outcomes even after controlling for socioeconomic variables. ^13^ Subsequent research showed that collective efficacy predicts collaborative instructional practice, sustained reform efforts, and resilience in the face of contextual challenges. ^15^ ^,^ ^21^ This body of work suggests that shared educator beliefs constitute a powerful determinant of educational effectiveness.

Medical education shares key features with school education, including interdependent teaching roles, shared responsibility for learner development, and coordinated assessment systems. However, while constructs such as faculty engagement, climate, and culture have been explored in health professions education, the explicit translation of collective efficacy theory to faculty groups has remained limited. The adaptation of this construct to medical education therefore addresses a theoretical and practical gap by focusing specifically on shared beliefs about collective educational capability. Extending these insights to medical education highlights the need to explicitly conceptualize how faculty groups perceive their collective capacity to influence educational processes and outcomes.

Taken together, these bodies of literature suggest that shared beliefs about collective capability play a central role in shaping educational outcomes, not only through individual performance but through coordinated group action.

### Defining collective educator efficacy in medical education

Collective Educator Efficacy (CEE) is conceptualized at the level of a defined faculty collective responsible for a coherent educational function, such as a course team, clerkship faculty group, residency program faculty, or departmental teaching unit. It is not an institutional climate measure nor an individual belief, but a shared perception within a bounded teaching group.

CEE refers to shared beliefs about the group’s capacity to influence both learner outcomes and program-level outcomes. Learner outcomes include knowledge acquisition, clinical competence, professional identity development, and progression through training. Program-level outcomes include curricular coherence, assessment quality, alignment with educational goals, and the ability to adapt to emerging challenges.

CEE spans multiple domains of faculty educational work, including didactic teaching, clinical supervision, assessment design and calibration, learner support and remediation, and curriculum coordination. Although these domains differ operationally, they share reliance on coordinated faculty action, justifying a unified construct that captures belief in collective educational impact.

Although CEE is conceptualized primarily at the level of a defined faculty collective (e.g., a course team, clerkship faculty, or residency program faculty), medical education programs often consist of multiple interdependent teams. Future work should therefore examine the relationship between local team-level CEE and a broader, program-level sense of collective efficacy. Program-level CEE would reflect shared beliefs about the overarching educational program’s capacity to achieve its aims, particularly in distributed training environments where coordination across sites and units is essential. These levels are likely interrelated but not reducible to one another, suggesting a multilevel structure of collective efficacy within medical education systems. To operationalize this construct, the core proposed dimensions of CEE, along with observable faculty behaviors and potential evaluation approaches, are outlined in Table 2 **.**

**Table 2.** Proposed dimensions of Collective Educator Efficacy (CEE) in medical education.

CEE dimension	Conceptual description	Observable faculty behaviours	Potential evaluation approaches
Shared educational vision	Faculty members hold a common understanding of educational goals, programme priorities, and learner development.	Consistent educational messaging, aligned teaching priorities, coordinated curriculum planning.	Faculty surveys, interviews, curriculum document analysis.
Collective confidence in educational impact	Faculty believe that coordinated action can meaningfully improve learner outcomes and programme quality.	Persistence during educational challenges, optimism regarding programme improvement, willingness to assume shared responsibility.	Group-referent efficacy scales, faculty focus groups.
Assessment and judgement efficacy	Faculty believe they can collectively make accurate, fair, and defensible educational judgements.	Active participation in calibration sessions, competency committee engagement, consistent application of standards.	Assessment calibration audits, observation of committee processes, survey measures.
Collaborative implementation capacity	Faculty believe they can effectively coordinate educational activities across courses, clerkships, or training sites.	Cross-disciplinary planning, coordinated supervision, implementation of shared educational initiatives.	Network analysis, meeting observations, implementation fidelity assessments.
Collective reflective learning	Faculty believe that collaborative reflection can improve educational practice and programme functioning.	Open discussion of educational problems, joint review of learner data, collaborative curriculum refinement.	Reflective practice audits, qualitative interviews, observation of faculty meetings.
Adaptive collective agency	Faculty believe they can respond effectively to educational change, uncertainty, and emerging programme challenges.	Innovation adoption, collaborative problem-solving, adaptation during accreditation or curriculum reform.	Longitudinal programme evaluation, implementation outcome measures, mixed-methods assessment.

*Note*: This table summarizes the proposed dimensions of Collective Educator Efficacy (CEE), illustrating how the construct can be conceptualized, recognized in faculty practice, and evaluated within medical education programmes. The dimensions are derived from the conceptual synthesis presented in this review and are intended to guide future empirical operationalization and validation.

### Positioning CEE within medical education theory

CEE intersects with, but remains distinct from, several established theoretical lenses in medical education. Organizational culture theory examines shared values and norms but does not specifically address collective beliefs about educational capability. Communities of practice emphasize learning through participation and identity formation ^9^ ^,^ ^10^ ^,^ ^33^ but do not focus on perceived collective agency to produce outcomes. Implementation science explores factors influencing the uptake of innovations but does not fully address the belief systems that determine whether faculty persist in implementing change. Programmatic assessment theory emphasizes integrated assessment systems ^22^ but does not explicitly account for the collective efficacy beliefs required for faculty to enact those systems consistently. Faculty engagement and burnout research address motivation and well-being but do not explicitly consider collective beliefs about impact.

CEE therefore contributes a psychological agency perspective that complements structural, cultural, and procedural frameworks by focusing on shared beliefs about collective educational influence.

Unlike organizational culture, which reflects shared values and norms, or faculty engagement, which reflects motivation and involvement, Collective Educator Efficacy specifically captures shared beliefs about the group’s capability to influence outcomes. This distinction is important, as beliefs about capability directly shape persistence, coordination, and response to challenges within complex educational systems. Collective educator efficacy is best understood as a group-level agency construct that complements, rather than replaces, existing theories of culture, climate and implementation. For example, psychological safety helps explain whether faculty feel able to speak up, question existing approaches and learn from mistakes, whereas collective educator efficacy addresses whether faculty believe they can, together, exert educational influence. Similarly, shared mental models and communities of practice describe how faculty coordinate understanding and action, but they do not directly capture the group’s confidence in its collective capacity to achieve educational goals. ^33^ Positioning CEE alongside these constructs strengthens the framework by linking faculty belief, faculty interaction and educational implementation. ^23^ The conceptual positioning of CEE relative to these related constructs is illustrated in Figure 2 **.**

**Figure 2.**
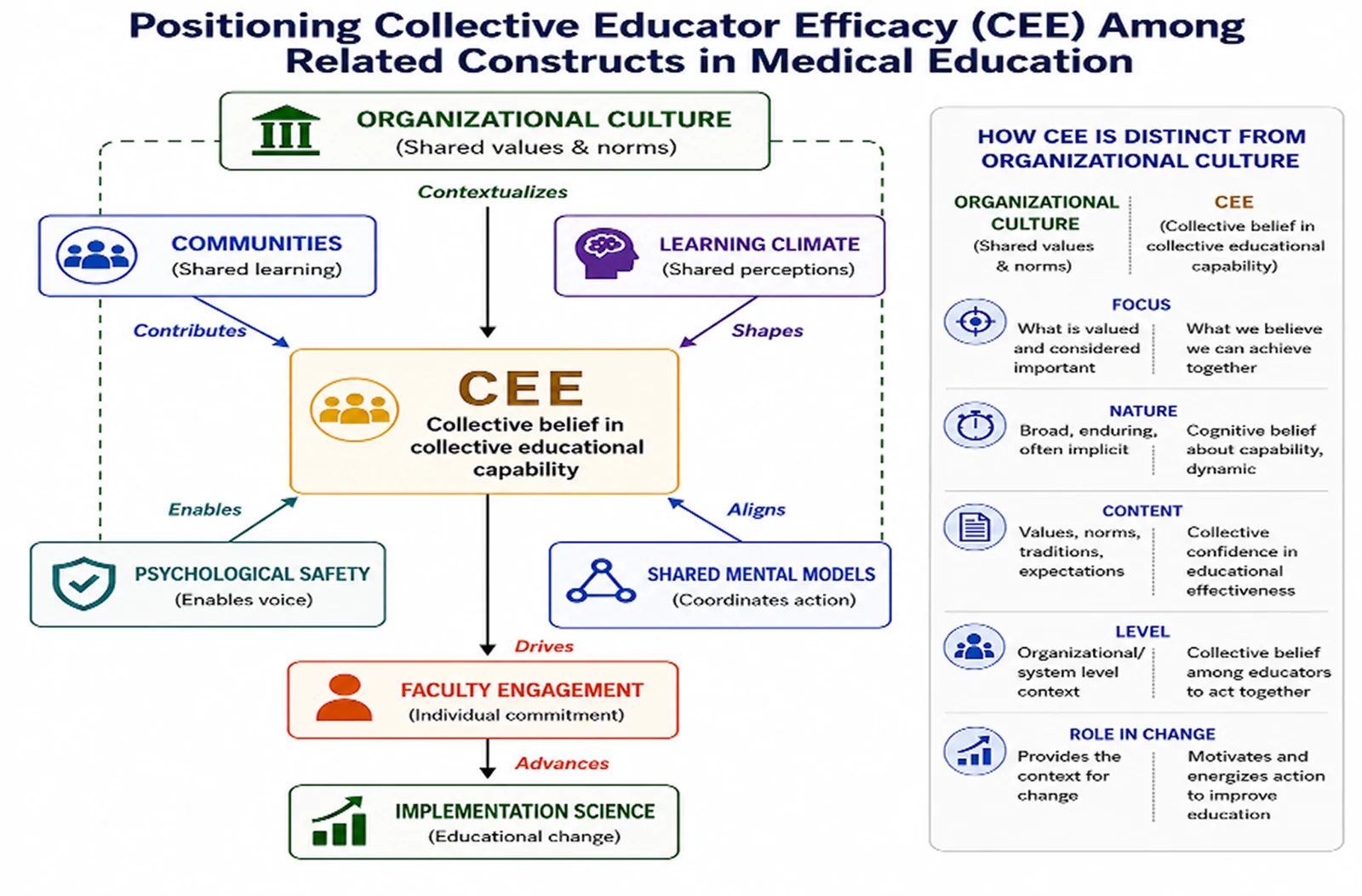
Conceptual positioning of Collective Educator Efficacy (CEE) within related theoretical constructs in medical education.

The framework illustrates how organizational culture provides the broader context, while communities of practice, learning climate, psychological safety, and shared mental models contribute to the development of CEE. CEE, in turn, drives faculty engagement and supports implementation science and educational change. The comparison panel highlights the conceptual distinctions between CEE and organizational culture.

### Why collective educator efficacy matters in medical education

Collective educator efficacy matters because medical education is delivered through coordinated faculty work. Curricula, assessment systems and feedback processes may be designed centrally, but their educational effect depends on how faculty groups enact them in local practice. Evidence from programmatic assessment shows that shared understanding between educators and learners is necessary for implementation, consensus statements on programmatic assessment emphasize operationalization and enabling conditions, and competency committee research demonstrates that group judgement quality depends on shared mental models, multiple perspectives and protection against bias. A CEE lens therefore offers a plausible explanation for why seemingly similar programs may differ in coherence, implementation fidelity, learner progression, and educational sustainability. ^6^ ^–^ ^9^ For example, integrated curricula demand alignment across disciplines. Faculty must coordinate sequencing, reinforce shared concepts, and prevent fragmentation. Without a shared belief that such coordination will meaningfully improve learner outcomes, faculty may default to siloed approaches. Similarly, competency-based medical education requires faculty to collectively uphold standards, contribute to programmatic assessment, and engage in shared decision-making about learner progression. These processes depend on trust in collective judgment and belief in the group’s ability to influence trainee development.

Assessment quality also relies on collective faculty engagement. Calibration, standard setting, and shared feedback practices require sustained collaborative effort. Faculty who believe their collective actions matter are more likely to invest time in these activities. Likewise, adoption of educational innovations from simulation integration to new assessment technologies depends on faculty believing that coordinated effort can produce improvement.

In these contexts, CEE functions as a motivational and organizational resource that shapes how faculty respond to complexity, change, and shared responsibility.

The absence of strong collective educator efficacy may also be diagnostically informative. In contexts of low CEE, faculty may comply with assessment requirements without engaging in meaningful calibration, leading to superficial standardization rather than genuine shared judgment. Faculty may express skepticism toward programmatic assessment data, viewing outcomes as reflections of learner deficits or external constraints rather than as information that the collective can use to improve. Such environments may be characterized by fragmented efforts, reduced willingness to invest in collaborative improvement initiatives, and a tendency to attribute educational challenges to factors beyond the group’s influence. Recognizing these behavioral patterns can help evaluators identify when limitations in collective belief, rather than structure alone, may be constraining program effectiveness.

## Discussion

### Proposed conceptual framework

We position Collective Educator Efficacy (CEE) as an emergent, group-level belief system operating within medical education programs understood as complex adaptive systems. In such systems, outcomes arise not from isolated curricular components but from the dynamic interactions among individuals, structures, and local contexts. ^21^ ^,^ ^24^ Within this perspective, CEE functions as a mechanism of collective agency that helps explain why programs with similar curricular designs, faculty qualifications, and resource profiles may nevertheless demonstrate strikingly different levels of implementation quality and learner outcomes. CEE does not describe morale or satisfaction; rather, it reflects the shared conviction among faculty that they can, through coordinated effort, influence educational processes and outcomes in meaningful ways.

The framework proposes that institutional conditions shape the development of CEE, that CEE in turn influences collective faculty behaviors, and that these behaviors ultimately affect program outcomes. Importantly, these relationships are dynamic and recursive rather than linear. Program outcomes feed back into faculty belief systems, strengthening or weakening collective efficacy over time.

Institutional conditions influence CEE through pathways consistent with social cognitive theory, which identifies mastery experiences, social persuasion, vicarious learning, and affective states as primary sources of efficacy beliefs ^11^ ^,^ ^25^ . Leadership practices play a central role in this process. Educational leaders who articulate a clear vision for teaching, legitimize educational work, protect time for coordination, and model collaborative problem-solving help faculty experience collective success. These experiences accumulate as evidence that coordinated action can produce meaningful change, thereby strengthening shared efficacy beliefs. In contrast, inconsistent priorities, fragmented decision-making structures, or environments in which teaching is devalued may erode collective confidence by repeatedly signaling that coordinated educational work is unlikely to succeed.

Faculty development structures constitute another important influence. When development initiatives are organized as collaborative endeavors such as faculty learning communities, shared curriculum redesign projects, or team-based assessment calibration, faculty gain opportunities to engage in collective mastery experiences. These activities do more than transmit individual teaching skills; they allow faculty to witness and participate in successful coordinated improvement, reinforcing beliefs in the group’s joint capability. This perspective aligns with scholarship arguing that faculty development should be viewed as an institutional strategy for culture-building rather than solely an individual capacity-building intervention. ^26^ ^,^ ^27^

Psychological safety further shapes the conditions under which CEE can emerge. When faculty perceive that their environment supports open dialogue, admission of uncertainty, and respectful disagreement, they are more likely to engage in the experimentation and candid reflection that generate collective learning. ^23^ Psychological safety is not synonymous with CEE, but it functions as enabling social infrastructure: without it, the collective mastery experiences necessary to build efficacy are less likely to occur.

Material and structural resources also communicate signals about collective possibility. Adequate time for coordination, access to assessment data, administrative support, and realistic workload expectations make coordinated educational work feasible. Chronic overload, lack of infrastructure, or repeated reform efforts without support may produce collective experiences of failure that weaken efficacy beliefs. Thus, institutional resource decisions can be understood not only as logistical determinants but also as psychological signals influencing faculty perceptions of what is achievable together.

Within this context, CEE influences program outcomes indirectly through its effects on collective faculty behaviors. Groups with stronger collective efficacy are expected to demonstrate more reliable coordination practices, greater persistence in collaborative tasks, and a stronger tendency to treat implementation challenges as solvable rather than inevitable. Such groups are more likely to develop shared mental models of competence, converging on common expectations regarding learner performance and progression. This shared understanding is critical in competency-based systems where distributed faculty must apply standards consistently.

CEE also supports engagement in assessment calibration and collective judgment processes. Activities such as standard setting, examiner training, and competency committee deliberation require sustained effort and trust in the value of collective decision-making. Faculty who believe that coordinated assessment work can meaningfully improve fairness and learner development are more likely to invest in these processes. Similarly, CEE is expected to foster collective reflection routines in which faculty regularly examine learner outcomes, interpret feedback data, and adjust curricula in response. Rather than viewing data as threatening, faculty with strong collective efficacy may interpret it as actionable information that the group can use to improve.

Adoption and sustainment of educational innovations represent another behavioral pathway. Implementation science has demonstrated that many innovations falter not because of flawed design but because of inconsistent execution and loss of momentum. Collective efficacy theory suggests that groups with strong shared beliefs in their joint capability are more likely to persist through the early setbacks and complexity associated with reform. ^10^ ^,^ ^11^ ^,^ ^22^ In this way, CEE may function as a sustainment resource that enables programs to carry innovations through initial resistance and operational challenges.

Through these behavioral mechanisms, CEE is expected to influence several program-level outcomes. Improved coordination and shared standards contribute to curricular coherence and continuity, reducing gaps and redundancies. Enhanced calibration and collective assessment practices improve consistency, validity, and defensibility of learner evaluation. ^22^ Stronger shared responsibility for learners may support more effective remediation processes and smoother learner progression. Finally, programs characterized by collective reflection and sustained innovation are likely to demonstrate greater adaptability in response to evolving accreditation requirements, clinical environments, and societal expectations.

A distinctive feature of this framework is its emphasis on feedback loops. Positive program outcomes provide collective mastery experiences that reinforce CEE, creating a virtuous cycle of confidence, coordinated action, and improvement. Conversely, repeated implementation failures, fragmented efforts, or chronic resource strain may generate shared experiences of ineffectiveness, weakening collective efficacy and contributing to downward trajectories. These dynamic processes help explain why programs can become “stuck” despite multiple structural reforms, as well as why others develop momentum that extends beyond any single intervention.

### Implications for program evaluation and faculty development

A CEE-informed evaluation design would extend conventional program evaluation by examining not only learner outcomes, but also faculty collective conditions that shape implementation. Relevant indicators would include perceived shared capability, assessment calibration, consistency of feedback practice, willingness to adapt teaching collectively, cross-site coherence, and the presence of psychologically safe dialogue around educational problems. These CEE-informed indicators can be integrated alongside conventional program evaluation measures, as summarized in Table 3. This approach aligns with contemporary program evaluation in health professions education, which argues that measuring outcomes alone is insufficient, and with faculty development scholarship showing that the impact of faculty development extends beyond individual skill acquisition to organizational change, collective practice and institutional culture. ^2^ ^,^ ^5^ ^,^ ^26^ ^,^ ^28^ ^,^ ^29^

**Table 3.** CEE-informed program evaluation matrix vs conventional indicators.

Evaluation Focus/Core Question	Conventional evaluation indicators	Added CEE-informed evaluation indicators
**Domain 1: Educational Processes & Implementation**		
**Consistency of Implementation** **(Is implementation consistent across faculty and clinical sites?)**	Lecture attendance rates Syllabus & curriculum completion logs Standardized examination scores	Perceived shared capability beliefs among faculty units Consensus on educational standards across sites Cross-site calibration frequency Collective faculty responsibility for implementation
**Feedback Quality & Alignment** **(Is feedback effective, actionable, and educationally aligned?)**	Learner satisfaction survey scores Recorded frequency of feedback encounters Completion rates of feedback forms	Psychological safety within teaching teams Faculty confidence in collaborative coaching Alignment and consistency of narrative feedback Shared reflective feedback culture
**Credibility of Assessment** **(Is assessment credible, defensible, and robust?)**	Learner progression & pass/fail statistics Psychometric reliability coefficients (e.g., Cronbach’s α) Objective examination score distributions	Shared mental models among assessment raters Multi-rater coherence across clinical rotation sites Routine collective calibration practices Cognitive bias mitigation in committee deliberations
**Translation of Faculty Development** **(Is faculty development translating into educational practice?)**	Workshop attendance logs Participation counts & CME credits issued Post-event participant satisfaction metrics	Shared implementation confidence across course teams Collaborative uptake of educational innovations Sustained collective behavioral change Faculty group willingness to engage in curricular reform
**Trustworthiness of Progression Decisions** **(Are learner progression decisions consistent and fair?)**	Competency committee decision counts Trainee remediation placement rates Graduation and board pass statistics	Shared interpretive frameworks for learner competence Inter-evaluator judgment consistency Faculty consensus regarding learner readiness for unsupervised practice
**Domain 2: Program Outcomes & Systems Adaptability**		
**Programmatic Adaptability** **(Can the program adapt to emerging educational challenges?)**	Frequency of formal curriculum revisions Policy updates and governance documentation Formal accreditation response reports	Collective faculty persistence through operational hurdles Collaborative problem-solving capacity Group willingness to challenge and refine existing practices Adaptive implementation capacity under stress
**Supportive Educational Climate** **(Is the educational climate conducive to learning?)**	Annual learner climate survey metrics Formal professionalism violation reports Incident reporting system counts	Mutual trust among clinical educators Collaborative teaching culture Educator psychological safety Perceived collective agency and openness to dialogue.
**Sustainability of Educational Reforms (Are educational innovations sustained over time?)**	Multi-year continuation of funded initiatives Administrative compliance reports Retention of formal policy mandates	Collective ownership of educational vision Shared commitment across department faculty Group resilience during leadership or organizational transitions Adaptive coordination across teaching teams
**Intended vs. Enacted Alignment** **(Is there alignment between intended and enacted curriculum?)**	End-of-course examination outcomes Curriculum mapping matrix logs Accreditation self-study documentation	Consistency between actual teaching practices and program goals Shared educational priorities across faculty cohorts Coordinated execution across teaching and clinical assessment teams

*Note*: Comparison of traditional outcome-focused program evaluation metrics against added CEE-informed indicators across educational processes and system adaptability domains.

Incorporating CEE into program evaluation expands the analytic lens beyond structural and outcome metrics to include the collective psychological conditions that influence how programs function in practice. Traditional evaluation approaches often focus on curricular design, learner performance, and individual teaching quality. ^3^ A CEE-informed approach asks an additional question: whether the faculty collective possesses the shared belief system necessary to execute the program reliably and adaptively. Differences in CEE may help explain why programs with similar resources and curricula display divergent outcomes. Measuring CEE could therefore provide a complementary diagnostic indicator, highlighting areas where faculty belief structures may be constraining effective implementation even when formal structures appear sound.

The framework also reframes faculty development. Rather than viewing development solely as a means of enhancing individual teaching skills, this perspective emphasizes the cultivation of collective capability. Faculty development initiatives that engage educators in shared problem-solving, collaborative curriculum work, and joint interpretation of learner data can generate the collective mastery experiences that build efficacy beliefs. ^26^ ^,^ ^27^ Leadership practices that promote trust, articulate shared goals, and support psychologically safe dialogue further contribute to this process. ^23^ ^,^ ^28^ ^,^ ^29^ In this view, strengthening CEE becomes an explicit objective of faculty development, linking individual learning with collective agency and program performance. The proposed integration of CEE-informed indicators into programme evaluation is summarized in Table 4 **.**

**Table 4.** Integrating collective educator efficacy (CEE) into program evaluation in medical education.

Evaluation question	Conventional indicators	Added CEE-Informed indicators
**Educational Processes**		
Is implementation consistent across faculty and sites?	Attendance rates, curriculum completion, examination performance	Faculty shared capability beliefs, consensus on educational standards, cross-site calibration, collective responsibility for implementation
Is feedback effective and educationally aligned?	Learner satisfaction scores, frequency of feedback encounters	Psychological safety, faculty confidence in collaborative coaching, consistency and alignment of narrative feedback, shared feedback culture
Is assessment credible and defensible?	Progression rates, psychometric reliability, pass/fail outcomes	Shared mental models among assessors, multi-rater coherence, collective calibration practices, bias mitigation in committee decisions
Is faculty development translating into educational improvement?	Workshop attendance, participation metrics, faculty satisfaction	Shared implementation confidence, collaborative uptake of innovations, sustained collective behavioural change, faculty willingness to engage in curricular improvement
Are learner progression decisions consistent and trustworthy?	Competence committee outcomes, remediation rates, progression statistics	Shared interpretive frameworks, collective judgement consistency, faculty consensus regarding learner competence and readiness
**Program Outcomes and Adaptability**		
Can the program adapt to emerging educational challenges?	Curriculum revisions, policy changes, accreditation responses	Faculty collective persistence, collaborative problem-solving capacity, willingness to challenge and improve practice, adaptive implementation capacity
Is the educational climate supportive of learning?	Learner climate surveys, professionalism reports, incident reporting	Faculty trust, collaborative culture, educator psychological safety, perceived collective agency, openness to reflective dialogue
Are educational reforms sustainable over time?	Continuation of initiatives, administrative reports, policy retention	Collective ownership, shared educational vision, resilience during organizational change, adaptive faculty coordination
Does the program maintain alignment between intended and enacted curriculum?	Examination outcomes, curriculum completion, accreditation reports	Consistency between faculty teaching practices and program goals, shared educational priorities, coordinated implementation across teaching teams

*Note*: This table illustrates how incorporating Collective Educator Efficacy (CEE) extends conventional program evaluation by adding faculty group level indicators that capture shared educational capability, coordinated implementation, and collective agency. The examples are conceptually derived from the present synthesis and are intended to guide future evaluation framework development rather than represent a validated measurement instrument.

### Operationalizing collective educator efficacy

Collective Educator Efficacy can be operationalized through both measurement and practice-based approaches within educational programs. At the measurement level, group-referent survey instruments can assess shared faculty beliefs regarding their collective ability to influence learner outcomes, assessment quality, and curriculum coherence. These measures can be adapted from existing collective efficacy scales and tailored to core educational tasks such as clinical supervision, competency committee decision-making, and curriculum coordination. Future validation should include group-referent item development, expert assessment of content validity, examination of dimensionality, and justification for aggregating individual responses to the faculty-group level. Convergent and discriminant validity should also be examined against related constructs such as collective efficacy, group potency, psychological safety, faculty engagement, and organizational culture.

At the practice level, CEE can be strengthened through structured faculty interactions, including assessment calibration sessions, competency committee deliberations, collaborative curriculum planning meetings, and faculty development initiatives that emphasize shared problem-solving. These activities provide opportunities for collective mastery experiences, which are central to the development of efficacy beliefs. Embedding such practices within routine program structures allows CEE to function as both a diagnostic indicator and a target for educational improvement. These processes align with the proposed framework by linking institutional conditions, collective faculty behaviors, and program outcomes through shared efficacy beliefs.

This conceptual synthesis is limited by the heterogeneity of the literature and the absence of empirical validation of CEE. The authors propose to examine its measurement properties, distinctiveness from related constructs, and applicability across medical education settings in future empirical work.

## Conclusions

Collective Educator Efficacy offers a conceptual lens for understanding variation in program effectiveness that cannot be fully explained by curricular design, faculty credentials, or institutional resources alone. By focusing on shared faculty beliefs about collective capability, CEE shifts attention from structural components of medical education to the psychological and relational mechanisms that shape how programs function in practice. Positioned within a systems perspective, CEE operates as a dynamic mediator between institutional conditions and educational outcomes, helping to explain why similarly designed programs may diverge in implementation quality and learner progression.

Faculty groups that believe in their collective capacity to influence learner development are more likely to engage in coordinated action, uphold shared standards, and sustain innovation in the face of complexity. Incorporating CEE into program evaluation and faculty development broadens the analytic toolkit of medical education by integrating psychological agency into structural and cultural frameworks. Future empirical work is needed to validate and operationalize this construct, but recognizing the role of collective educator beliefs is an important step toward more system-informed approaches to strengthening educational programs.

## Data Availability

No new datasets were generated or analyzed in this narrative review.
