# Supplementary material for: Collective Educator Efficacy in Medical Education: A Conceptual Synthesis and Framework for Program Evaluation
Source: MedEdPublish (2016). 2026 Sep 12;16:87. [Version 1] doi: 10.12688/mep.21825.1 (PMC13736064; doi:10.12688/mep.21825.1)
Supplement: Peer review history

# Reviewer report: Reviewer response for version 1

**DOI:** 10.21956/mep.23374.r46934

**Published:** 2026-11-04

**Authors:** Sara Reza, Alia Hussain

**Affiliations:** 1 Quaid-e-Azam Medical College, Bahawalpur, Pakistan; 2 Mid and South Essex NHS Trust, England, England, UK

We found the article interesting because it shifts the focus of medical education programme evaluation beyond curriculum design and learner outcomes to faculty teams’ shared belief in their ability to improve education.

 The review defines Collective Educator Efficacy (CEE) as a group’s shared belief that it can achieve educational goals together, highlighting it as an underexamined influence on programme implementation and quality. Drawing on Bandura’s theory of collective efficacy and research on collective teacher efficacy, the authors present a conceptual model (Figure 1), distinguish CEE from related constructs (Figure 2), outline six dimensions (Table 2), and propose CEE-informed evaluation indicators (Tables 3 and 4).

 I also find the central question especially compelling: why do structurally similar programmes sometimes perform so differently? Shared faculty beliefs at the group level offer a promising, often-overlooked explanation, and the paper is clear and engaging.

 However, I have a few reservations. These concerns are set out below in order of importance.

 1. Distinctiveness of the construct

 CEE closely parallels collective teacher efficacy. Fives and Looney (ref. 16) have already examined collective efficacy among college instructors. The authors' claim that medical education has not articulated such a construct would be more compelling if they demonstrated that they reviewed related literature in HPE, nursing, and healthcare teams, and clearly explained what CEE contributes beyond a change in setting.

 I support the previous reviewer’s recommendation to compare CEE with other collective constructs. The most significant omission is group potency. Although the authors cite Stajkovic et al.’s meta-analysis comparing collective efficacy and group potency (ref. 17), they do not explain how CEE relates to either construct. A concise comparison table could address this issue by covering CEE, collective efficacy, group potency, team efficacy, and psychological safety.

 2. Reporting of methods

 The methods describe searches of six databases, dual screening, and third-reviewer adjudication. However, the authors report no numbers: records identified, screened, excluded at each stage, or reasons for exclusion. Some points need clarifying:

 •    Search window: the window is January 1990 to May 2026, but included sources include Bandura 1977 (ref 25). Several included sources are books (refs 11, 30, 33) that would not be retrieved from the listed databases. Please specify which sources came from database searching and which from citation tracking or the authors’ prior knowledge.

 •    Size of the included set: The 30 included sources appear to make up the whole reference list apart from the three methodology papers.

 •    Framing: It may be more accurate to frame the work as a conceptual or critical review with purposive sampling, rather than present a systematic-style search that is only partly reported.

 3. Accuracy of citations

 I agree with the previous reviewer that references need verifying.

 •    Ref 2 (school inclusion and student involvement) supports a statement about systems perspectives in health professions education (Introduction) and is listed under psychological safety in Table 1. A medical education source would be more appropriate.

 •    Refs 9 and 10 (programmatic assessment; Bandura on collective efficacy) are cited to characterise communities of practice.

 •    Ref 12 (Bleakley, on complexity) is cited with Bandura as a source for collective efficacy theory (“10–12”), including the sources of efficacy beliefs.

 •    Ref 21 (Donohoo et al., collective efficacy) supports the claim that outcomes in complex adaptive systems arise from continuous interactions.

 •    Ref 22 (van der Vleuten, 1996, assessment of professional competence) is cited for implementation-science claims about persisting through reform setbacks.

 •    Ref 13: the third author should be Woolfolk Hoy A, not “Woolfolk HA.”

 •    Figure 1 legend: the citations use author–date format (Bandura, 1997; Goddard et al., 2000; Tschannen-Moran & Barr, 2004), unlike the numbered style used elsewhere.

 4. Boundaries of the framework

 Table 2 and the proposed evaluation indicators seem to mix beliefs about what faculty can achieve together with things they actually do, such as assessment calibration, curriculum coordination, and shared decision-making. These are related, but they are not quite the same. It would help to separate CEE itself from the conditions that support it, the behaviours that may follow from it, and the eventual programme outcomes. This would also make the framework easier to test in future studies.

 5. Measurement and interpretation

 The authors mention group-referent surveys, but how would individual faculty responses be combined to represent a shared belief? Would some agreement within the group be needed before calling it collective efficacy? A little more detail would be helpful here.

 The paper also occasionally gives the impression that CEE already explains differences in programme outcomes. As this is still a proposed framework, I would suggest using more cautious language and making it clear which links are supported by existing evidence and which remain to be tested. It may also be worth acknowledging that strong collective confidence is not always beneficial; it could sometimes encourage groupthink or resistance to feedback.

 6. Minor points

 •    The definition of CEE in the Introduction is missing a subject (“…that, through coordinated effort, [they] can positively influence…”).

 •    The definition in the Abstract differs from the one in the Introduction; one definition should be used consistently.

 •    Spelling alternates between “program” and “programme”; one should be used throughout.

 •    SANRA: SANRA is a quality-appraisal scale, not a reporting guideline. The wording “reported with attention to SANRA domains” could be adjusted.

 •    The section on low CEE as diagnostically informative is entirely uncited and should be framed as conjecture.

 •    Tables 3 and 4 overlap considerably and could be combined. This would make the practical implications easier to follow.

 Overall

 This bright conceptual contribution could inspire useful empirical work. Citation and methods-reporting issues are fixable; the main challenge is clarifying the construct’s boundaries and distinguishing it from its antecedents and consequences to make the framework testable. We would welcome a revised version.

Is the review written in accessible language?

Yes

Are all factual statements correct and adequately supported by citations?

Partly

Are the conclusions drawn appropriate in the context of the current research literature?

Partly

Is the topic of the review discussed comprehensively in the context of the current literature?

Yes

Reviewer Expertise:

NA

We confirm that we have read this submission and believe that we have an appropriate level of expertise to confirm that it is of an acceptable scientific standard, however we have significant reservations, as outlined above.

# Reviewer report: Reviewer response for version 1

**DOI:** 10.21956/mep.23374.r46768

**Published:** 2026-10-22

**Authors:** Amira Farghaly

**Affiliations:** 1 Basic Medical Sciences, Prince Sattam bin Abdulaziz University College of Medicine, Al-Kharj, Riyadh Province, Saudi Arabia

I thank the authors for a thoughtful and timely paper. Translating collective efficacy to faculty groups in medical education is a genuine contribution. The discussion of programmatic assessment and competency committees is particularly well argued.

 My main concern is how the paper frames program evaluation, the field it aims to extend. The manuscript does not define program evaluation or name any evaluation model. It also characterizes evaluation as mainly outcome-focused, although several widely used models explicitly evaluate context, inputs and process. As a result, the gap CEE is said to fill is overstated. The comments below are meant to help the authors make the contribution narrower but more credible.

 - The title, abstract, keywords and main argument all centre on program evaluation, but the term is never defined. I recommend adding a definition early in the Introduction.

 - The manuscript refers to "standard evaluation frameworks", "conventional program evaluation" and "traditional evaluation approaches". However, it names no model. Kirkpatrick's four levels, the Logic Model, CIPP, and experimental or quasi-experimental designs are all absent. So are participatory or empowerment evaluation, theory-driven evaluation and contribution analysis.

 - The Abstract states that program evaluation "often emphasizes outcomes". The Introduction says it "traditionally focuses on learner outcomes" and "rarely examines the collective psychological and cultural conditions of the faculty". This is accurate for some models, but not for evaluation as a field. For example, the CIPP model and the logic model focus on the inputs and processes in addition to outcomes. 

 - In Tables 3 and 4, conventional evaluation is reduced to counts and outcomes, such as attendance logs, CME credits and pass rates. A CIPP Process study or a well-built Logic Model would already capture several of the "added CEE-informed indicators". Examples include implementation consistency, barriers encountered, participants' role-taking and the uptake of faculty development. I suggest the conventional column reflect what process-oriented models actually examine. The added column could then focus on what is genuinely new: shared beliefs about collective capability.

 - The manuscript repeatedly states that CEE "explains" variation between similar programs, and that low CEE "constrains" effectiveness. These are causal claims about a construct that has not yet been measured in medical education. I suggest more tentative wording ("may help explain"). The authors could also note how attribution might be examined later.

 - The CEE framework shows only positive pathways. The authors should consider possible downsides of high collective efficacy. Examples are overconfidence, groupthink in competency committees, and resistance to external feedback or reform.

 - Tables 3 and 4 largely duplicate each other. They share the same evaluation questions and nearly identical indicators. I suggest keeping one, or giving the second a distinct purpose.

 - The search and selection process needs more detail. Six databases searched over 36 years yielded 30 included sources, but no screening numbers are reported. Even for a narrative review, the numbers identified, screened and excluded would improve transparency. The SANRA domains the authors cite also ask for this.

Is the review written in accessible language?

Yes

Are all factual statements correct and adequately supported by citations?

Partly

Are the conclusions drawn appropriate in the context of the current research literature?

Partly

Is the topic of the review discussed comprehensively in the context of the current literature?

Partly

Reviewer Expertise:

Medical Education, Curriculum Development, Student Assessment, Program Evaluation

I confirm that I have read this submission and believe that I have an appropriate level of expertise to confirm that it is of an acceptable scientific standard, however I have significant reservations, as outlined above.

# Reviewer report: Reviewer response for version 1

**DOI:** 10.21956/mep.23374.r46687

**Published:** 2026-09-23

**Authors:** Shivasakthy Manivasakan

**Affiliations:** 1 Sri Balaji Vidyapeeth, Puducherry, India

The Collective Educator Efficacy provides a useful conceptual lens for understanding the key factors that influence educational outcomes. The narrative review has included 30 references in this context. However, as a limitation of the narrative review, the authors acknowledged that empirical validation and operationalisation are still required. A 2025 systematic review and meta-analysis (not medical education, though) is a good reference in the same context. https://doi.org/10.1016/j.tate.2025.105006

 The authors can improve the comprehensibility of the manuscript by comparing the CEE with other collective constructs. 

 The search strategy can be expanded with more details on the methodology.

 References and in-text citations need to be verified. Reference 2 can be replaced with a similar reference from medical education. More references from HPE can add value to the manuscript. The conclusion strength can be moderated to suit the narrative review context.

Is the review written in accessible language?

Yes

Are all factual statements correct and adequately supported by citations?

Yes

Are the conclusions drawn appropriate in the context of the current research literature?

Yes

Is the topic of the review discussed comprehensively in the context of the current literature?

Yes

Reviewer Expertise:

Health Professions Education

I confirm that I have read this submission and believe that I have an appropriate level of expertise to confirm that it is of an acceptable scientific standard.
